# Inflammatory Mediators in Tracheal Aspirates of Preterm Infants Participating in a Randomized Trial of Inhaled Nitric Oxide

**DOI:** 10.1371/journal.pone.0169352

**Published:** 2017-01-03

**Authors:** Mandy Laube, Elena Amann, Ulrike Uhlig, Yang Yang, Hans W. Fuchs, Michael Zemlin, Jean-Christophe Mercier, Rolf F. Maier, Helmut D. Hummler, Stefan Uhlig, Ulrich H. Thome

**Affiliations:** 1 Center for Pediatric Research Leipzig, Hospital for Children & Adolescents, Division of Neonatology, University of Leipzig, Leipzig, Germany; 2 Division of Neonatology and Pediatric Critical Care, Department of Pediatrics, University of Ulm, Ulm, Germany; 3 Institute of Pharmacology and Toxicology, RWTH Aachen University, Aachen, Germany; 4 Department of Pediatrics, University Medical Center Freiburg, Freiburg, Germany; 5 Department of Pediatrics, University of Marburg, Marburg, Germany; 6 Department of Pediatrics, University of Saarland, Homburg, Germany; 7 Hôpital Universitaire Robert Debré, Paris, France; Medical University of South Carolina, UNITED STATES

## Abstract

**Background:**

Ventilated preterm infants frequently develop bronchopulmonary dysplasia (BPD) which is associated with elevated inflammatory mediators in their tracheal aspirates (TA). In animal models of BPD, inhaled nitric oxide (iNO) has been shown to reduce lung inflammation, but data for human preterm infants is missing.

**Methods:**

Within a European multicenter trial of NO inhalation for preterm infants to prevent BPD (EUNO), TA was collected to determine the effects of iNO on pulmonary inflammation. TA was collected from 43 premature infants randomly assigned to receive either iNO or placebo gas (birth weight 530–1230 g, median 800 g, gestational age 24 to 28 2/7 weeks, median 26 weeks). Interleukin (IL)-1β, IL-6, IL-8, transforming growth factor (TGF)-β_1_, interferon γ-induced protein 10 (IP-10), macrophage inflammatory protein (MIP)-1α, acid sphingomyelinase (ASM), neuropeptide Y and leukotriene B_4_ were measured in serial TA samples from postnatal day 2 to 14. Furthermore, TA levels of nitrotyrosine and nitrite were determined under iNO therapy.

**Results:**

The TA levels of IP-10, IL-6, IL-8, MIP-1α, IL-1β, ASM and albumin increased with advancing postnatal age in critically ill preterm infants, whereas nitrotyrosine TA levels declined in both, iNO-treated and placebo-treated infants. The iNO treatment generally increased nitrite TA levels, whereas nitrotyrosine TA levels were not affected by iNO treatment. Furthermore, iNO treatment transiently reduced early inflammatory and fibrotic markers associated with BPD development including TGF-β_1_, IP-10 and IL-8, but induced a delayed increase of ASM TA levels.

**Conclusion:**

Treatment with iNO may have played a role in reducing several inflammatory and fibrotic mediators in TA of preterm infants compared to placebo-treated infants. However, survival without BPD was not affected in the main EUNO trial.

**Trial registration:**

NCT00551642

## Introduction

Survival of preterm infants is frequently associated with a chronic lung disease called bronchopulmonary dysplasia (BPD). Infants who develop BPD often require long-term oxygen supplementation [1,2] and frequent re-admissions to hospitals [3,4], resulting in high health care costs [5]. Quality of life may be reduced, especially since pulmonary function abnormalities may persist into adulthood [6,7].

Lung injury induced by mechanical ventilation and oxygen supplementation triggers pro-inflammatory responses and repair processes [8–10]. Cytokines can be measured in tracheal aspirates (TA) and reflect the extent of inflammatory reactions [11,12]. It was reported that interleukin (IL)-1, IL-6, IL-8, intercellular adhesion molecule-1 (ICAM-1), macrophage inflammatory protein (MIP)-1α, transforming growth factor (TGF)-β_1_ and leukotriene B_4_ (LTB_4_) were increased within the first 10 days of life in the bronchoalveolar lavage (BAL) fluid of preterm infants who later developed BPD as compared to those who did not [13–19]. In a multicenter high frequency oscillatory ventilation trial [20], the outcome of the trial was predicted by IL-8 and LTB_4_ TA levels [21]. One further important chemokine in lung injury that is critical for leukocyte activation is CXCL10 (IP-10) [22]. Another potentially interesting mediator is neuropeptide Y (NPY), because it may be involved in the so-called neuro-immune axis [23]. In addition, increased levels of glycolipids, such as ceramide, were found in the BAL fluid of patients with respiratory distress syndrome (RDS) [24] and in an ovine BPD model [25]. Furthermore, acid sphingomyelinase (ASM), an enzyme generating ceramide, was increased in ovine BPD [25] and in septic patients, where it correlated with their mortality [26]. These findings are remarkable, because the ASM pathway is critical for edema formation in many models of acute lung injury [27] including neonatal piglet models [28,29] and may also promote apoptosis in lung epithelial cells [30,31].

Nitric oxide (NO) is a gaseous mediator that–apart from its vasodilatatory properties–has various effects on inflammation [32,33] and vascular endothelial growth factor (VEGF)-mediated tissue remodeling [34], both possibly modifying the development of BPD. In animal models of BPD, inhaled NO (iNO) has been shown to reduce lung inflammation, apoptosis and oxidative stress, to maintain surfactant activity, and to improve lung structure and alveolarization [33,35–41]. In humans, a randomized trial demonstrated that iNO reduced the incidence of BPD without increasing the risk of intracranial bleeding in mechanically ventilated preterm infants [42]. However, subsequent studies did not observe similar beneficial effects [43–45]. Aside from possible benefits, NO is a free radical, potentially capable of causing oxidative tissue damage. Furthermore, it might combine with superoxide to form peroxynitrite, a powerful nitrating and tissue damaging substance [46]. Reactive oxygen and nitrogen species, such as peroxynitrite, also participate in lung injury [47,48]. Although they cannot be measured directly because of their instability, peroxynitrite formation can be estimated from the resulting nitration products, especially nitrated tyrosine residues on various proteins including surfactant protein A [49,50]. On the other hand, iNO may alleviate free radical toxicity, as NO has been shown to decrease lipid peroxidation and spare α-tocopherol by scavenging peroxy radicals [51,52].

Within a European multicenter trial of NO inhalation for preterm infants to prevent BPD (EUNO trial) [45], TA were collected at two study centers to determine the effects of iNO on pulmonary inflammation. We hypothesized that iNO reduces pro-inflammatory and pro-fibrotic cytokines and ASM in TA. Furthermore, we hypothesized that iNO may be associated with a higher amount of nitrite and nitrotyrosine possibly indicating increased peroxynitrite formation.

## Materials and Methods

### The EUNO trial

In brief, infants with a gestational age between 24 and 28 2/7 weeks were enrolled if they weighed at least 500 g and required surfactant or continuous positive airway pressure for RDS within 24 h of birth. Treatment was initiated within 2 h of enrollment, but not later than 26 h of life. Infants were randomized to receive either iNO (5 parts per million [ppm]) or placebo gas (nitrogen gas) for a minimum of 7 days and a maximum of 21 days in a double-blind fashion [45]. BPD at 36 weeks’ postmenstrual age was defined by the physiological criteria of Walsh and colleagues [53,54]. Infants enrolled at two of the study centers (Marburg and Ulm, Germany) were eligible for TA sampling if they were endotracheally intubated for mechanical ventilation.

### Tracheal aspirate sampling

This sub-study was specifically approved by the institutional review board (INOT27, reference number 220/2004, Ulm, Germany) which also oversaw the main trial. Written informed parental permission was obtained specifically for the sub-study (collection of TA samples), in addition to permission for enrollment in the study. After initiation of iNO or placebo gas treatment TA was collected during normal medically indicated endotracheal suctioning procedures on postnatal days (PD) 2, 4, 7, 14 and 21, unless the infant was extubated earlier. By protocol, infants also received the randomized gas at least until they were weaned off of ventilatory support or reached PD21. Herein, we report the TA data obtained between PD2 and PD14, since increasing numbers of infants were extubated within the study period resulting in a low statistical power for data obtained on PD21. If less than 4 specimens per sampling day were obtained, further specimen were collected on the following day. No samples were collected within 4 hours of a surfactant instillation. A standardized procedure was used. For sampling, a sterile mucus trap was inserted in the suctioning system, and 1 ml/kg birth weight of normal saline was instilled into the endotracheal tube, and the ventilator briefly reconnected (3–5 breaths). The suction catheter was then flushed with 0.5 ml normal saline. TA was transferred to an appropriate tube and immediately centrifuged with 140 x g at 4°C for 10 minutes. The supernatant was transferred to cryotubes and frozen immediately below -20°C. Within 72 hours, cryotubes were transferred to a -80°C freezer and held at -80°C until ready for shipment to the laboratory, which was done on dry ice.

### Tracheal aspirate analyses

All analyses were performed at the Institute of Pharmacology and Toxicology of the RWTH Aachen (Aachen, Germany). For the parameters IL-1β, IL-6, IL-8, IP-10 and MIP-1α a Bio-Plex Cytokine assay (Bio-Rad Laboratories, Munich Germany) was used [55]. Enzyme-linked immunosorbent assays (ELISA) were used for albumin (# EA2201-1; AssayPro; St. Charles, USA), nitrotyrosine (# HK501; Cell Sciences; Canton, USA), and TGF-β_1_ (# DB100B; R&D Systems GmbH, Wiesbaden-Nordenstadt, Germany). Furthermore, competitive binding assays were employed for LTB_4_ (# KGE006B; R&D Systems GmbH) and NPY (# EK-049-03; Phoenix Europe GmbH, Karlsruhe, Germany). Nitrite concentration was analyzed using a Griess reaction assay (# KGE001; R&D Systems GmbH). All assays were performed according to the manufacturer’s recommendations. For ASM, a proprietary assay was used [56]. Samples from the same patient and day were pooled to increase the amount and decrease variations of dilution. No attempt to normalize data was made since no uniformly accepted standard is currently available. We expressed the data per milliliter of TA as recommended by the European Respiratory Task Force on Bronchoalveolar Lavage in children [57,58] and reported by others [59,60].

### Statistical analyses

Demographic and clinical outcome data were compared between iNO- and placebo-treated groups by Mann Whitney U test or Fisher’s Exact test as appropriate using GraphPad Prism (version 6.05; GraphPad Software, Inc, San Diego, CA, USA). Measured TA concentrations were compared by mixed model two-way (factors being time and treatment) analyses of variance (ANOVA) with a heterogeneous first-order autoregressive covariance structure using SAS software 9.4 (GLIMMIX procedure, SAS Institute, Cary, NC). Post-tests were performed for the treatment effect on each day and *p*-values were adjusted for multiple comparisons by the simulated Shaffer procedure. In the figures, the effect of time is denoted below the x-axis, the overall effect of treatment to the right of the graphs, and the treatment effects on each single day at the respective time points.

## Results

During the recruitment period from the years 2006–2008, 67 infants were enrolled in the original EUNO trial in the two study centers. Of these 67 infants, 49 infants were recruited for this sub-study. No TA could be obtained from 6 infants, because of extubation within the first 24 h of life in 4 infants and missing parental consent for TA sampling in 2 infants, resulting in 43 infants available for TA sampling. Of these 43 infants, 25 received iNO and 18 placebo gas. Demographic data were similar between the iNO- and placebo treated groups (Table 1). Clinical sepsis was defined by the maximal C-reactive protein (CrP) exceeding 20 mg/L within the first 72 h of life. Blood cultures were negative for all infants. Mean airway pressures and FiO_2_ represent maximum values observed before initiation of iNO or placebo gas treatment.

**Table 1:** Demographic characteristics of the infants.

	iNO	Control	*p*-values
Number of patients	25	18	
Gestational age (weeks)*	26 (24 1/7-28 2/7)	26 1/7 (24–28 2/7)	0.812
Birth weight (g)*	800 (530–990)	815 (630–1230)	0.142
Male	14 (56.0%)	11 (61.1%)	0.765
Race (white)	25 (100%)	18 (100%)	1.000
Prenatal steroids	24 (96%)	18 (100%)	1.000
Apgar score 5-min*	8 (2–10)	9 (3–10)	0.633
Apgar score 10-min*	9 (7–10)	10 (5–10)	0.400
Surfactant replacement therapy	20 (80%)	16 (88%)	0.680
Death	1 (4%)	1 (6%)	1.000
BPD	5 (20.8%)	2 (11.8%)	0.679
Duration of ventilation (days)*	47 (9–141)	39 (17–82)	0.209
Therapy duration (days)*	21 (8–22)	21 (11–22)	0.605
Maternal chorioamnionitis	11 (44.0%)	11 (64.7%)	0.223
PPROM	5 (20.0%)	5 (29.4%)	0.717
Duration of PPROM (h)*	133 (23–600)	148 (38–878)	0.841
Mean airway pressures*	7.5 (0.8–26)	9 (5–28)	0.081
Sepsis	3 (12%)	5 (29.4%)	0.247
FiO_2_*	0.6 (0.31–1)	0.6 (0.28–1)	0.820

*Median (minimum-maximum), Mann Whitney U test; all others: Fisher’s exact test.

PPROM: preterm prolonged rupture of membrane; FiO_2_: fraction of inspired oxygen.

**Fig 1:**
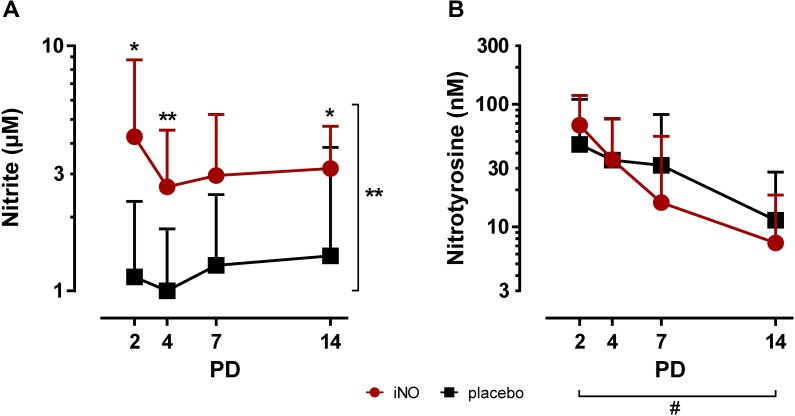
Nitrite and nitrotyrosine TA concentrations in infants treated with iNO compared to placebo-treated controls. Serial TA samples were obtained from PD2 to PD14. Data are displayed as the mean of TA levels and SD on a logarithmic scale. **A:** Nitrite TA levels were overall increased by iNO treatment (⋆⋆, p<0.01) and specifically elevated on PD2 (⋆, p<0.05), PD4 (⋆⋆, p<0.01) and PD14 (⋆, p<0.05) in iNO-treated infants. **B:** Nitrotyrosine TA levels were not altered by iNO treatment and decreased with increasing postnatal age of the infants from PD2 to PD14 (#, p<0.05). PD: postnatal day.

TA levels were compared by mixed model ANOVA (Table 2) determining the effect of iNO treatment and postnatal age on the analyzed variables. Furthermore, the effect of iNO treatment was analyzed for each measured time point from PD2 to PD14. Nitrite levels in TA samples of iNO-treated infants were significantly higher over the complete study period (p<0.01; Fig 1A). In particular, nitrite TA levels were significantly elevated by iNO on PD2 (iNO: 4.26 ± 4.52 μM [mean ± SD] versus control: 1.14 ± 1.18 μM; p<0.05), PD4 (iNO: 2.66 ± 1.87 μM versus control: 1.0 ± 0.79 μM; p<0.01) and PD14 (iNO: 3.16 ± 1.54 μM versus control: 1.39 ± 2.46 μM; p<0.05). Postnatal age did not affect nitrite TA levels which were constant over the study period. In contrast, nitrotyrosine TA concentrations were not different between iNO-treated infants and the placebo-treated control group, whereas nitrotyrosine levels decreased significantly over the study period in both groups (p<0.05; Fig 1B). On PD7 and PD14 the majority of TA samples were negative for nitrotyrosine in both groups.

**Table 2:** Mixed model ANOVA (*p*-values).

	iNO treatment	Postnatal age
Nitrotyrosine	p = 0.6468	**p = 0.0312**
Nitrite	**p = 0.0028**	p = 0.9801
ASM	p = 0.1117	**p<0.0001**
TGF-β_1_	**p = 0.0224**	p = 0.0575
IP-10	p = 0.3126	**p<0.0001**
IL-1β	p = 0.8465	**p<0.0001**
IL-8	p = 0.352	**p = 0.0202**
IL-6	p = 0.9856	**p = 0.0407**
MIP-1α	p = 0.7646	**p<0.0001**
Albumin	p = 0.9069	**p = 0.0073**
LTB_4_	p = 0.9153	p = 0.3528
NPY	p = 0.5156	p = 0.8368

*P*-values of the main factor of the two-way ANOVA: treatment and time (postnatal age). None of the interaction effects was significant.

**Fig 2:**
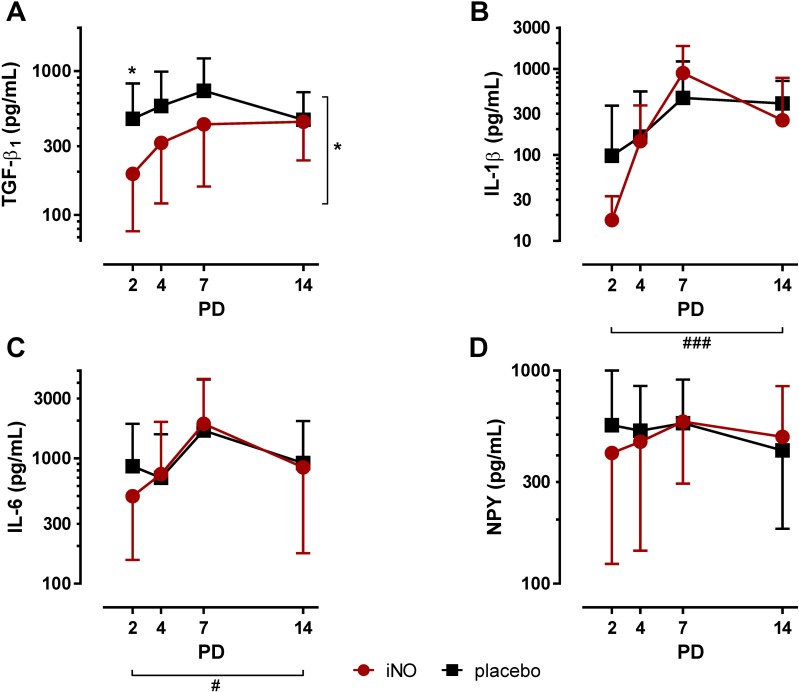
Cytokine TA concentrations in infants treated with iNO compared to placebo-treated controls. Serial TA samples were obtained from PD2 to PD14. Data are displayed as the mean of TA levels and SD on a logarithmic scale. **A:** TGF-β_1_ TA levels were overall decreased by iNO treatment (⋆, p<0.05) and specifically decreased on PD2 (⋆, p<0.05) in iNO-treated infants. **B:** IL-1β TA levels were not significantly affected in iNO-treated infants and increased with advancing postnatal age from PD2 to PD14 in both groups (###, p<0.001). **C:** IL-6 TA levels were not altered by iNO treatment and increased with advancing postnatal age from PD2 to PD14 in both groups (#, p<0.05). **D:** NPY TA levels were not altered by iNO treatment or postnatal age. PD: postnatal day.

TGF-β_1_ TA concentrations were significantly decreased by iNO on PD2 (iNO: 193 ± 116 pg/ml versus control: 466 ± 353 pg/ml; p<0.05; Fig 2A). Furthermore, TGF-β_1_ levels were lower in iNO-treated infants over the entire study period (p<0.05; Fig 2A). Postnatal age did not significantly affect TGF-β_1_ TA levels. IL-1β TA levels were not significantly affected by iNO treatment (Fig 2B). However, postnatal age strongly affected IL-1β TA levels, which significantly increased from PD2 to PD14 in both groups (p<0.001; Fig 2B). IL-6 TA concentrations were not affected by iNO treatment, but significantly increased with advancing postnatal age in both groups (p<0.05; Fig 2C). NPY levels, in contrast were altered neither by postnatal age nor by iNO treatment (Fig 2D).

**Fig 3:**
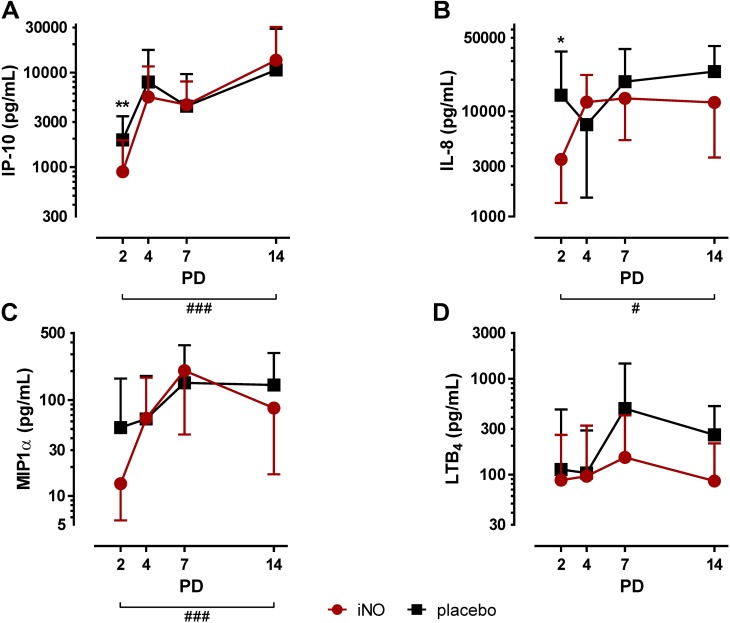
Chemokine TA concentrations in infants treated with iNO compared to placebo-treated controls. Serial TA samples were obtained from PD2 to PD14. Data are displayed as the mean of TA levels and SD on a logarithmic scale. **A:** IP-10 TA levels were significantly decreased on PD2 in iNO-treated infants (⋆⋆, p<0.01) and increased with advancing postnatal age from PD2 to PD14 in both groups (###, p<0.001). **B:** IL-8 TA levels were significantly decreased on PD2 in iNO-treated infants (⋆, p<0.05) and increased with advancing postnatal age from PD2 to PD14 in both groups (#, p<0.05). **C:** MIP-1α TA levels were not affected by iNO treatment and increased with advancing postnatal age from PD2 to PD14 in both groups (###, p<0.001). **D:** LTB_4_ TA levels were not altered by iNO treatment or postnatal age. PD: postnatal day.

Among the chemokines, IP-10 TA levels were significantly decreased by iNO treatment on PD2 (iNO: 892 ± 1,028 pg/ml versus control: 1,940 ± 1,501 pg/ml; p<0.01; Fig 3A). On PD4, PD7 and PD14 no differences for IP-10 were observed between the study groups. Postnatal age strongly affected IP-10 TA levels which significantly increased from PD2 to PD14 (p<0.001). In addition to TGF-β_1_ and IP-10, IL-8 TA concentrations were significantly reduced by iNO on PD2 with 3,485 ± 2,140 pg/ml in the iNO group compared to 14,262 ± 22,931 pg/ml in the placebo-treated control group (p<0.05; Fig 3B). No difference for IL-8 was observed on PD4, PD7 and PD14 between iNO-treated infants and the placebo-treated control group. Furthermore, IL-8 TA levels significantly increased from PD2 to PD14 in both groups (p<0.05). MIP-1α TA levels were not significantly affected by iNO, but strongly increased in both, iNO- and placebo-treated infants from PD2 to PD14 (p<0.001, Fig 3C). In contrast, LTB_4_ TA levels were neither affected by postnatal age nor by iNO treatment (Fig 3D).

**Fig 4:**
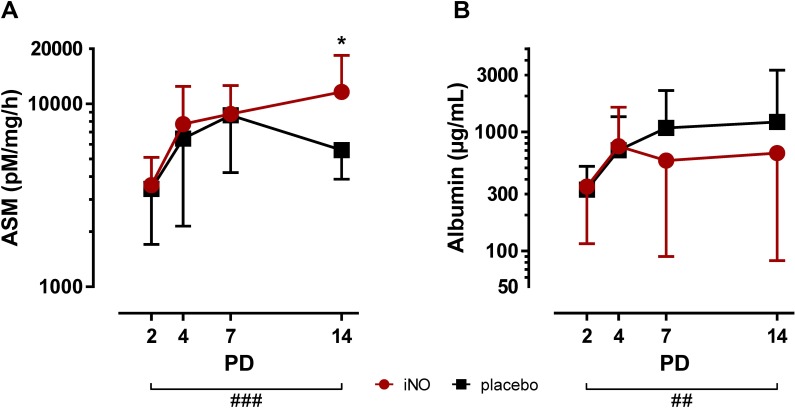
ASM and albumin TA concentrations in infants treated with iNO compared to placebo-treated controls. Serial TA samples were obtained from PD2 to PD14. Data are displayed as the mean of TA levels and SD on a logarithmic scale **A:** ASM TA levels were significantly elevated on PD14 in iNO-treated infants (⋆, p<0.05) and increased with advancing postnatal age from PD2 to PD14 in both groups (###, p<0.001). **B:** Albumin TA levels were not altered by iNO treatment and increased with advancing postnatal age from PD2 to PD14 in both groups (##, p<0.01). PD: postnatal day.

ASM TA concentrations were similar between iNO- and placebo-treated infants on PD2, PD4 and PD7, but the iNO group showed elevated ASM activities on PD14 with 11,631 ± 6,822 pM/mg/h compared to the placebo-treated control group with 5,589 ± 1,713 pM/mg/h (p<0.05; Fig 4A). Moreover, ASM TA levels strongly increased over the study period from PD2 to PD14 in both groups (p<0.001). Finally, albumin TA levels were not affected by iNO treatment as no significant differences were observed between the study groups at PD2 to PD14, but advancing postnatal age significantly elevated albumin TA levels over the study period (p<0.01; Fig 4B).

In conclusion, iNO treatment increased the nitrite and ASM concentrations in TA samples of preterm infants at individual time points, but had no effect on nitrotyrosine TA levels. Furthermore, TGF-β_1_ TA levels were generally lower in iNO-treated infants, and IP-10 and IL-8 TA concentrations were transiently reduced by iNO treatment on PD2. Postnatal age, affected almost every analyzed TA variable, demonstrated by significant effects on nitrotyrosine, ASM, IP-10, IL-1β, IL-8, IL-6, albumin and MIP-1α concentrations.

## Discussion

Inflammation is crucially involved in the development of BPD and results from an imbalance between pro- and anti-inflammatory mediators [61]. Mechanical ventilation triggers the pulmonary influx of neutrophils and macrophages that produce a variety of cytokines and other signalling molecules [61]. Herein, we sought to determine the effect of iNO treatment on pulmonary inflammatory mediators in TA of preterm infants as part of the EUNO trial [45]. Our study showed that iNO treatment of preterm infants significantly reduced early TA levels of TGF-β_1,_ IP-10 and IL-8. While the effect of iNO on TGF-β_1_ persisted throughout the study, the effects on IP-10 and IL-8 were transient only. Furthermore, nitrite TA levels were increased by iNO treatment throughout the study period and ASM levels were increased after 2 weeks. To our knowledge, only one study has analyzed the impact of iNO treatment on pulmonary inflammatory mediators in preterm infants. In contrast to our results, this study found no significant changes in TA concentrations of IL-8, IL-1β or TGF-β_1_ induced by iNO [62]. These discrepancies might be due to the different study design, since we started iNO treatment within the first 24 h of life, employing low-dose iNO, whereas the study of Truog and colleagues started iNO treatment at PD7 with 20 ppm iNO. Most iNO effects observed in our study occurred early and transiently at PD2 and might not be detectable once the inflammatory response is already established.

NO is rapidly oxidized *in vivo* and the concentration of its metabolites, mainly nitrite and nitrate serve as biomarkers for NO [63]. Hence, iNO has been shown to dose-dependently affect nitrite and nitrate concentrations in TA and plasma, thereby demonstrating an effective delivery of iNO to the lung and the systemic circulation [64]. In line with this, we observed elevated TA levels of nitrite at PD2 and PD4, which remained elevated until PD14. In the presence of superoxide, NO may form peroxynitrite, which nitrates the phenolic residues of tyrosine, forming nitrotyrosine. We did not observe an increased nitrotyrosine TA concentration in the iNO group, and nitrotyrosine levels similarly decreased in both groups during the study period, which may be related to the diminishing exposure to supplemental oxygen with increasing postnatal age. A previous study showed that plasma nitrotyrosine concentrations were elevated during the first month of life in infants who developed BPD [65]. Another study on the therapeutic effects of iNO demonstrated that preterm infants whose nitrotyrosine levels decreased within the first 72 h of life were more likely to wean off of mechanical ventilation [66]. In agreement, another trial using iNO for preterm infants did not detect any changes of plasma nitrotyrosine levels induced by iNO, nor was the nitrotyrosine concentration altered in infants with BPD compared to infants without BPD [67,68].

Early increases of pro-fibrotic TGF-β_1_ in TA have been demonstrated in preterm infants that subsequently developed BPD [14], which was most pronounced on PD2 to PD4 [14,60]. Therefore, an increase of TGF-β_1_ may represent an early event in the process leading to BPD that precedes abnormalities in lung function due to tissue remodeling and fibrosis (see review [69]). We demonstrated significantly lower TGF-β_1_ TA levels in iNO-treated infants from PD2 on. Most important for the preterm lung, TGF-β_1_ has been shown to inhibit epithelial cell maturation and the synthesis of phospholipids and surfactant proteins A, B and C in human fetal lung explants [70]. In agreement, one study demonstrated a transiently improved surfactant function in preterm infants undergoing iNO treatment [71]. TGF-β_1_ activates fibroblast differentiation into myofibroblasts [72] and induces alveolar and bronchial epithelial cells to undergo epithelial-mesenchymal-transition [73,74], which contributes to interstitial thickening and fibrosis *in vivo* [75]. Notably, exogenous NO has been shown to attenuate epithelial-mesenchymal-transition induced by TGF-β_1_ in alveolar epithelial cells [76] and iNO therapy has been shown to reduce hyperoxia-induced fibrin deposition and septal thickening in rat pups [77]. Interestingly, TGF-β_1_ has recently been shown to inhibit β_2_-adrenergic receptor-mediated fluid transport across rat alveolar type II cell monolayers and alveolar fluid clearance (AFC) *in vivo* by down-regulation of β_2_-adrenergic receptors [78,79]. AFC is crucially involved in resolution of pulmonary fluid at birth and a TGF-β_1_-mediated AFC reduction possibly contributes to prolonged ventilator dependence of preterm infants leading to the development of BPD. Therefore, a reduction of early TGF-β_1_ levels in preterm infants at risk of developing BPD by iNO treatment might suggest a beneficial clinical outcome.

Our study further showed that iNO treatment reduced the TA levels of different pro-inflammatory cytokines. First, the downstream target of IFN-γ, the chemokine IP-10 showed increasing TA concentrations during the study period in both groups, yet iNO treatment significantly reduced IP-10 levels at PD2, compared to the placebo-treated group. In a study of ventilated preterm infants, the IFN-γ and IP-10 TA levels were higher within the first 48 h of life in infants that developed BPD or died [80,81], suggesting that increases in IFN-γ and IP-10 levels precede neutrophil infiltration and could therefore represent critical early response molecules in the development of BPD [82], similar to what has been suggested for ARDS [22]. Thus, the early reduction of IP-10 levels by iNO might indicate a diminished inflammatory status in those infants that might be accompanied by a reduced recruitment of inflammatory cells to the lung. This is supported by an experimental BPD model of mechanically ventilated premature lambs in which low-dose iNO (5 ppm) decreased early lung neutrophil recruitment and accumulation [33]. However, the effect of iNO on IP-10 levels was transient. In addition, increased IL-8 TA levels have been described on PD1 and PD3 in preterm infants who subsequently developed BPD, preceding the neutrophil infiltration [83–85]; however, elevated TA IL-8 levels also seem to be associated with BPD development in older infants [13,86]. Here we found a rise of IL-8 TA concentrations during the study period and iNO treatment significantly reduced IL-8 TA levels at PD2. Since early IL-8 TA elevations supposedly precede neutrophil infiltrations in BPD risk infants [83], an early reduction induced by iNO might reduce neutrophil recruitment to the lung. In agreement with our results, iNO treatment has been shown to reduce BAL IL-8 concentrations and neutrophil infiltration in a pig model of lung injury [87] and human patients with ARDS [88]. Similar to IP-10, the effect of iNO on IL-8 levels was transient. The reason for this observation is currently unknown, but decreasing NO absorption or an elevated first-pass effect seems unlikely, since nitrite TA levels were relatively stable over the study period. Because infants are extubated as soon as possible, thus limiting TA sampling, a selection towards the more serious cases or patients in which iNO treatment failed to improve inflammatory mediator levels possibly contributes to the early transient effects of iNO. Another possible reason for the transient effect of iNO might be due to the development of tolerance to the clinical and physiological effects of NO upon continuous administration [89]. The mechanism of tolerance to NO donors has been thoroughly investigated, especially in the treatment of cardiovascular diseases, but remains mainly elusive and highly debated. Notably, tolerance to nitrovasodilators generally begins to develop within 24 to 48 h of continuous application [89], and could thus be an explanation for the early transient effects of iNO observed in our study. Next-generation NO donors with improved pharmacokinetic properties might be able to prolong the anti-inflammatory effects of NO treatment in critically ill preterm infants.

We further observed increased ASM TA activities in iNO-treated infants on PD14. Although the function of circulating ASM is presently unknown, elevated ASM levels were found in different respiratory disorders (see reviews [27,90]), and we demonstrated increasing ASM activities throughout the current study in critically ill infants of both groups. Currently, it is unknown if BPD development in preterm infants is associated with ASM activity or ceramide levels. Many agonists, including tumor necrosis factor (TNF)-α and platelet activating factor (PAF) stimulate ceramide production [90] and PAF-induced pulmonary edema is partly mediated by ASM and ceramide [56]. On the other hand, inhibition of the ASM pathway as well as the *de novo* synthesis pathway of ceramide was shown to elevate IL-8 production in TNF-α stimulated respiratory epithelial cells, suggesting that ASM and ceramide may be involved in terminating an ongoing IL-8 production [91]. By contrast, ceramide has been shown to trigger IL-1β release in a mouse model of acute lung injury [92]. Yet, IL-1β TA levels were not elevated by iNO at PD14, suggesting that the up-regulation of ASM TA activities did not elevate the level of inflammatory mediators analyzed in our study. Finally, IL-6, albumin, LTB_4_ and NPY TA levels were not significantly altered by iNO treatment compared to placebo-treated infants, but in contrast to LTB_4_ and NPY, IL-6 and albumin TA levels increased throughout the study in both groups.

Several clinical trials have evaluated whether iNO reduces the mortality or the incidence of BPD in preterm infants with sometimes contradictory results [42,67]. The EUNO trial demonstrated that the early use of low-dose iNO (5 ppm) in very premature infants did not improve survival without BPD [45]. Inconsistencies between different iNO trials may be due to differences in dose, duration, age at treatment initiation, study population and/or other factors [93]. Therefore, results of these clinical trials are not conclusive and the use of iNO treatment for preterm infants to prevent BPD is currently not recommended [94]. Notably, a multicenter, randomized trial comparing high-frequency oscillatory ventilation with conventional ventilation in the early treatment of respiratory disease in very preterm infants showed no difference for the primary endpoint death or BPD incidence [95]. However, the follow-up of this trial demonstrated superior lung function at 11 to 14 years of age in former infants assigned to high-frequency oscillatory ventilation [96]. This suggests a beneficial outcome, although BPD incidence did not differ in the initial trial. With regard to this long term outcome, follow-up results for iNO therapy are currently awaited and BPD incidence possibly constitutes an imprecise endpoint to predict clinical benefits.

A limitation of this study was the small sample size, increasing the likelihood of statistical errors. Moreover, because in clinical practice infants are extubated as soon as feasible to limit potential damage from continuing ventilation, the number of infants of whom TA could be obtained declined with increasing postnatal age. This results in fewer TA samples and hence lower statistical power for TA analyses of infants older than one week. Furthermore, because of the limited sample size not all mediators could be measured from each TA sample. Therefore, subsequent trials with larger groups are required to confirm these results. We further did not include clinical characteristics such as maternal chorioamnionitis, PPROM, ventilator parameters, FiO_2_ requirements or sepsis as confounding variables in our analyses, which may play a role in release of inflammatory mediators. In addition, it is controversial whether TA should be corrected for dilution, using techniques such as albumin content, urea or secretory immunoglobulin A concentrations. Currently, no uniformly accepted correction factor to normalize cytokine TA levels exists, which impedes comparison of results obtained by different normalization procedures. As recommended by the European Respiratory Task Force on Bronchoalveolar Lavage in children [57,58], we did not correct our results for the dilution during TA sampling, and expressed our data per milliliter of TA.

## Conclusions

In conclusion, the study showed that the TA levels of IP-10, IL-6, IL-8, MIP-1α, IL-1β, ASM and albumin increased with advancing postnatal age in critically ill preterm infants, whereas nitrotyrosine TA levels declined in both iNO-treated and placebo-treated infants. Furthermore, iNO treatment strongly increased nitrite TA levels throughout the study period. Besides, a delayed increase of ASM TA levels induced by iNO was detected. A beneficial effect of iNO was demonstrated on early inflammatory/fibrotic markers including TGF-β_1_, IP-10 and IL-8. Whether the demonstrated reduction of early inflammatory mediators possibly improves long-term lung function has to be determined in follow-up studies, since BPD incidence was not affected by iNO treatment in the EUNO trial [45].
